# The contribution of the left precuneus to emotion memory in migraine without aura patients

**DOI:** 10.3389/fnins.2022.905942

**Published:** 2022-10-18

**Authors:** Meiqin Li, Xiaoshu Li, Wanqiu Zhu, Jiajia Zhu, Haibao Wang, Ziwen Gao, Xingqi Wu, Shanshan Zhou, Kai Wang, Yongqiang Yu

**Affiliations:** ^1^The First Affiliated Hospital of Sun Yat-sen University, Guangzhou, China; ^2^The First Affiliated Hospital of Anhui Medical University, Hefei, China

**Keywords:** migraine without aura, emotional memory, magnetic resonance imaging, gray matter volume, resting-state functional connectivity

## Abstract

**Background:**

The impact of migraine without aura (MWoA) on cognitive function remains controversial, especially given the sparse literature on emotional memory.

**Methods:**

Twenty seven MWoA patients and 25 healthy controls (HCs) were enrolled in this cross-sectional study. Emotional memory behavior was evaluated by combining incidental encoding with intentional encoding of five emotional categories of visual stimulus [positive valence + high arousal (PH), negative valence + high arousal (NH), positive valence + low arousal (PL), negative valence + low arousal (NL), and neutral (N)]. The recollection performance (Pr) was measured and compared. Then, the neural relevance was explored by correlating the Pr with gray matter volume (GMV) and resting-state functional connectivity (rs-FC) based on structural and functional magnetic resonance imaging.

**Results:**

No significant differences in recollection performance or emotional enhancement of memory effect were observed. However, MWoA patients were more sensitive to the valence and arousal of emotional stimuli under incidental encoding. Significantly, the Pr-PH under incidental encoding and Pr-PL under intentional encoding were negatively correlated with the GMV of the left precuneus, and the rs-FC between the left precuneus and putamen was positively correlated with Pr-PL under intentional encoding in MWoA patients.

**Conclusion:**

Our study demonstrated the tendency for the influence of migraine on emotional memory and revealed the left precuneus as a critical contributor to recollection performance, providing novel insights for understanding emotional memory and its neural mechanisms in MWoA patients.

## Introduction

Migraine is a common neurological condition, with a global prevalence of approximately 15% (Headache Classification Committee of the International Headache Society, 2018). Migraine without aura (MWoA) is the most subtype classified by the International Headache Society (Silberstein et al., 2005). As a disabling headache disorder, mechanisms involved in MWoA remain unclear, and the neuropsychological impairment remains controversial (Tardiolo et al., 2019). Some studies found that several cognitive tests are unaffected by migraines (Le Pira et al., 2000; Gaist et al., 2005; Pearson et al., 2006; Camarda et al., 2007; Pellegrino Baena et al., 2018). In a population-based study of Danish twins, the cognitive performance of the twins with MWoA did not differ from non-migraineurs, and comparisons within twin pairs yielded comparable results (Gaist et al., 2005). Moreover, a retrospective single-blinded study reported that cognitive functions remained unimpaired even with a long history of MWoA (Pearson et al., 2006). However, other studies found that MWoA might lead to poor cognitive performance in executive function, processing speed, attention, and memory (Le Pira et al., 2000; Camarda et al., 2007; Pellegrino Baena et al., 2018). Such indeterminate conclusions need further exploration and research, particularly in less frequently studied high-order cognitive functions, such as emotional memory.

Previous studies have established that emotional events were better recollected than non-emotional events, a phenomenon known as the emotional enhancement of memory (EEM) effect (LaBar and Cabeza, 2006). Emotional memory paradigms were implemented to investigate the EEM effect with features such as arousal and valence (Kensinger, 2004). Arousal dichotomizes excitement and calmness, and stimuli with high arousal can enhance the initial encoding and subsequent consolidation of events by attracting attention (Mather and Sutherland, 2011). In contrast, valence refers to the positive or negative aspects of emotional stimuli that enhance memory (Dolcos and Cabeza, 2002). Memory recall from pictures or words with negative valence produces potent effects compared to positive valence, suggesting that memory may favor negative stimuli (Inaba et al., 2005; Mickley and Kensinger, 2008; Bowen and Kensinger, 2017; Bowen et al., 2018; Farris and Toglia, 2019). Studies conducted on emotional memory in Alzheimer’s disease found that emotional memory was impaired, and the EEM effect was lost (Li et al., 2016). However, young adults with migraine have been reported to have a higher risk for dementia (Chuang et al., 2013). Moreover, the pathophysiology of dementia began approximately 20 years before the onset of clinical symptoms (Sperling et al., 2014). Besides, it has been shown that the prevalence of white matter hyperintensities in migraine is 38.7 ∼ 44.4% (Dobrynina et al., 2021), and the incidence of subclinical brain infarction was twice that of healthy controls (HCs) (Monteith et al., 2014). Thus, this study aims to investigate whether there is emotional memory damage in MWoA patients and further explore the underlying neural mechanism by correlating the recollection performance with voxel-wise gray matter volume (GMV) and resting-state functional connectivity (rs-FC) values, considering that the cerebral cortex and FC are crucial for brain functions (Shafiee et al., 2020; Wirsich et al., 2020).

## Materials and methods

### Participants and settings

This cross-sectional study recruited 32 right-handed MWoA patients from the headache clinic of The First Affiliated Hospital of Anhui Medical University in China between June 2018 and February 2019. MWoA diagnoses were based on the headache characteristics and the International Classification of Headache Disorders 3rd edition (ICHDIII criteria) (Headache Classification Committee of the International Headache Society, 2018). Patients included in this study were18 to 60 years of age with a migraine history of 1 year before the study, experiencing a minimum of one attack per month with moderate-to-severe pain 3 months before screening. The exclusion criteria were as follows: head trauma or vascular disease; previous or current psychiatric or neurological disorders or somatoform disorders, such as depression, stroke, and dementia; substance abuse; anerythrochloropsia; magnetic resonance imaging (MRI) contraindications. Thirty-one sex-matched and age-matched volunteers from the community in the same geographical area with no personal or family history of migraine were recruited as HCs for this effort. The exclusion criteria for MWoA were also applied to HCs. Migraineurs and healthy subjects were diagnosed and screened by a specialist headache neurologist. Five MWoA patients and six HCs were excluded for analysis due to technical issues with the MRI data (motion artifact) or emotional memory data (recording issues), resulting in a final sample of 27 MWoA patients and 25 HCs.

General demographic information, including age, sex, and education level, was collected from all participants. Montreal Cognitive Assessment (MoCA), Hamilton Anxiety Scale (HAMA), and Hamilton Depression Scale (HAMD) were used to evaluate global cognitive function, anxiety, and depression, respectively. The emotional memory test and MRI scans were performed during the inter-migraine period, a 2-day interval during the absence of acute migraine attacks. The characteristics of migraine, such as duration of disease, monthly frequency and duration of attacks, and pain intensity/impact, were collected.

### Emotional memory behavioral test

#### Stimulus

**FIGURE 1:**
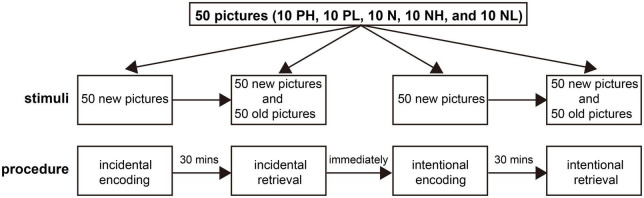
The procedure of the emotional memory paradigm.

Two hundred pictures were selected from the International Affective Picture System (Lang et al., 2010), a widely used collection of color photographs featuring the two independent emotional properties of arousal and valence. Both properties ranged from 1 to 9, where 1 corresponded to a very negative valence or low arousal state, and 9 corresponded to a very positive or high arousal state. The valence was considered negative, positive, and neutral for ratings of ≤ 4, ≥ 6.0, and 4.5–5.5, respectively. Similarly, the arousal was considered high, low, and neutral for ratings of ≥ 6.0, ≤ 4.0, and 4–6, respectively. According to valence and arousal ratings, the pictures were divided into five categories: positive with high arousal (PH), positive with low arousal (PL), neutral (N), negative with high arousal (NH), and negative with low arousal (NL) (Li et al., 2020). The neutral category excluded high/low arousing and valence images. These pictures were depicted in 2 lists of 100 stimuli. Then each list was divided into two 50-picture subgroups. Each subgroup consisted of 10 PH, 10 PL, 10 N, 10 PL, and 10 PL. For each list, one subgroup (50 pictures) was presented during the encoding phase, and both subgroups (100 pictures—50 seen and 50 unseen) were presented during the retrieval phase (Figure 1). These pictures included humans and landscapes and were presented in counterbalance.

#### Apparatus

The E-prime v.2.0 software (PST Inc., Sharpsburg, PA, USA) was used to present the stimuli and record the participants’ responses on a laptop computer.

#### Procedure

The procedure began when the participants were ready and acclimatized to the new environment, a room for neuropsychological testing. The behavioral test was divided into incidental and intentional sessions according to encoding. Each session had an encoding phase, where participants were asked to immediately identify whether the main object shown in a picture was a person (categorization task). During this phase, the stimulus was presented for 2,000 ms, and the stimulus interval was 500 ms. Then, there was a 30 min delay between the encoding and retrieval phases. During the retrieval phase, the remember/know procedure was employed to estimate recollection and familiarity directly (Yonelinas, 1994). Participants were asked to identify whether the image presented was old (seen during the encoding phase) or new (or not). If the pictures were considered old, they were then asked whether they recollected the details of the images (recollection) or were only familiar with the pictures (familiarity) based on their memory differences (recognition task) (Quamme et al., 2010). Some quality control methods were performed to ensure that each subject could distinguish recollection from familiarity as accurately as possible and follow the same criteria. Practice examples were provided with on-screen instructions before the test to ensure that the participants understood each task and the difference between recollection and familiarity. Moreover, participants were asked to describe the criteria they used in the retrieval task at the end of the test. During the incidental session, participants were unaware of the later retrieval task in the encoding phase. However, participants were asked to memorize the pictures carefully when encoded during the intentional session. The intentional session began after the completion of the incidental session (Figure 1). Investigators and participants were double-blinded for the test.

### Magnetic resonance imaging acquisition

All MRI data were acquired on a General Electric 750 w 3.0 T MRI scanner (General Electric, Waukesha, WI, USA) with a 24-channel head coil. The MRI protocol included the acquisition of three-dimensional T1-weighted (3D T1) high-resolution structural images, resting-state blood oxygen level-dependent scans, and axial T2-weighted and FLAIR images. The included participants did not show structural abnormalities on the MRI examination. The BRAVO (brain volume) sequence [repetition time (TR) = 8.5 ms, inversion time (TI) = 450 ms, echo time (TE) = 3.2 ms, 188 slices, no slice gap, slice thickness = 1 mm, field of view (FOV) = 256 × 256 mm^2^, matrix size = 256 × 256, and flip angle = 12°] was used to acquire the 3D T1 images. An echo-planar imaging sequence (TR = 2,000 ms, TE = 30 ms, slice gap = 1 mm, slice thickness = 3 mm, FOV = 220 × 220 mm^2^, matrix size = 64 × 64, and flip angle = 90°) was used to acquire resting-state functional MRI (rs-fMRI) scans.

#### Structural magnetic resonance imaging pre-processing

Structural 3D T1 images were pre-processed using the VBM8 (voxel-based morphometry)^1^ toolbox in SPM8 (Statistical Parametric Mapping)^2^ in Matlab (Mathworks, Natick, MA). First, we visually inspected all structural images to screen for anatomical abnormalities or artifacts. The standard brain templates were used to segment the image into gray matter (GM), white matter (WM), and cerebrospinal fluid (CSF) volumes. A diffeomorphic anatomical registration algorithm (DARTEL) toolbox was used to co-register structural MR images. For each subject, a flow field was created for wrapping scans onto the template. Then, gray matter images were normalized spatially to the Montreal neurological institute (MNI) coordinates using these data. Subsequently, the images were resampled at 1.5 × 1.5 × 1.5 mm voxel size and smoothed with an 8 mm full-width half-maximum (FWHM) Gaussian kernel to construct a DARTEL template (Cheng et al., 2015).

#### Resting-state functional magnetic resonance imaging data pre-processing

The rs-fMRI data were pre-processed with DPABI (Data Processing and Analysis of Brain Imaging)^3^ (Yan et al., 2016), a MatLab toolbox. First, we accounted for the magnetic field instability, and the initial 10 volumes were removed for each scan. Then, images within each scan were realigned using 1.5 mm and 1.5° movement thresholds to correct motion between time points. The frame-wise displacement was calculated, and other covariates, such as estimated motion parameters and the WM and CSF signals, were regressed. The data set was then bandpass filtered between 0.01 and 0.08 Hz. Finally, the individual structural images were co-registered to the mean functional image. Then, the structural and functional images were normalized to the MNI space using the DARTEL toolbox. These images were resliced to a 3 × 3 × 3 mm voxel and spatially smoothed with a 6 mm FWHM Gaussian kernel. The DPABI software was used to define a seed region using automatic anatomical labeling (AAL) (Tzourio-Mazoyer et al., 2002), and the functional connectivity (FC) was calculated between the seed region and the rest of the brain. The correlation coefficients (r) were transformed into Fisher z-scores to obtain normally distributed values.

### Statistical analysis

#### Demographic data analysis

This was the primary analysis of the data. Statistical analysis was performed using SPSS 23.0 software package (SPSS, Chicago, III). Normally and skewed distributed variables were reported as mean ± standard deviation and median (25th, 75th percentiles), respectively. We assessed normality and compared general demographic characteristics between the two groups using a χ^2^-test for sex and Mann-Whitney *U*-tests for age, education level, MoCA, HAMA, and HAMD scores. Statistical significance was set at a two-tailed *p*-value < 0.05.

#### Emotional memory behavioral test

The hit (Hit) and false alarm (FA) rate was calculated as the ratio of the sum of remember and know judgments given to old and new pictures, respectively. This study used the difference between Hit and FA judged by memory as the recall score index, expressed as Pr (Koen and Yonelinas, 2016). *A priori* power analysis was performed using G*Power 3 program to compute the necessary sample size. Alpha, effect size, and power (1 – β) were set at 0.05, 0.25, and 80%, respectively (Cona et al., 2015). The sample size of 25 individuals in each group was considered sufficient. A 5 × 2 mixed-factorial covariance analysis was performed on Pr with the stimuli emotional categories (5 categories, PH, PL, N, NH, and NL) as the within-subject factors and the participant groups (2 groups) as the between-subject factors with the MoCA, HAMA, and HAMD scores as covariates. The Bonferroni correction was used for *post hoc* multiple comparisons. Investigators responsible for data analyses were blinded to patient grouping.

#### Gray matter volume analysis

Voxel-wise analysis of within-group correlation in GMV and Pr was conducted with a multiple regression model. Sex, age, education level, total intracranial volume, MoCA, HAMA, and HAMD scores were used as covariates.

#### Functional connectivity analysis

The significant brain region identified using correlation analyses between GMV and Pr in MWoA patients was used as the seed region in the rs-FC analysis. Voxel-wise analysis was performed on the within-group correlation with the rs-FC values and Pr using a multiple regression model. Sex, age, education level, head motion, MoCA, HAMA, and HAMD scores were set as covariates.

In GMV and FC analysis, we set the statistical threshold at *p*-value < 0.05 (cluster level, FWE corrected) with an extent threshold of 50 voxels. Significant clusters were automatically identified using the Xjview toolbox.^4^ Each significant cluster in GMV or rs-FC correlation analysis was extracted for each subject and used for the region of interest analysis.

## Results

### General demographic results

Table 1 contains demographic and clinical information of the study participants. There were no significant differences in gender, age, and education degree between the two groups (*p* > 0.05). In MWoA patients, the MoCA score was lower, and the HAMA and HAMD scores were higher than HCs (*p* < 0.05).

**TABLE 1:** General demographic information of participants.

	MWoA (*n* = 27)	HC (*n* = 25)	*p*
**Demographic characteristics**			
Age (years)	28 (25, 36)	28 (23, 38.5)	0.324
Sex (male/female)	6/21	10/15	0.165
Education degree (years)	16 (9, 17)	16 (12, 17)	0.899
**Cognitive**			
MoCA	28 (25, 28)	28 (27, 29)	*
**Emotion**			
HAMA	5 (1, 10)	1 (0.5, 2)	*
HAMD	6 (1, 10)	1 (0, 2)	*
**Migraine characteristics**			
Duration of migraines in years	10 (6, 14)	——	——
Monthly frequency of migraine attacks	4 (3, 4)	——	——
Attack duration	12 (12, 12)	——	——
NRS	5 (6, 7)	——	——
HIT-6	62 ± 7.1	——	——

*p < 0.001. The monthly frequency of migraine attacks was the mean frequency of 3 months before the participation interview. Pian intensity was calculated as the mean numeric rating scale (NRS: 0 = no pain to 10 = unbearable pain) score for the days with a headache. HIT-6, headache impact test, ranges from 36 to 78; a higher score represents a more severe headache.

### Emotional memory behavioral test results

#### Incidental encoding

**FIGURE 2:**
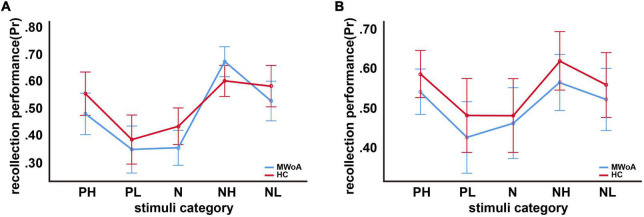
Recollection performance as a function of picture type. **(A)** Incidental encoding, **(B)** intentional encoding. Note that Pr represents recollection performance, which varies from 0 (no discrimination between old and new pictures) to 1 (perfect discrimination). Bars represent estimated marginal means ± standard deviations.

**FIGURE 3:**
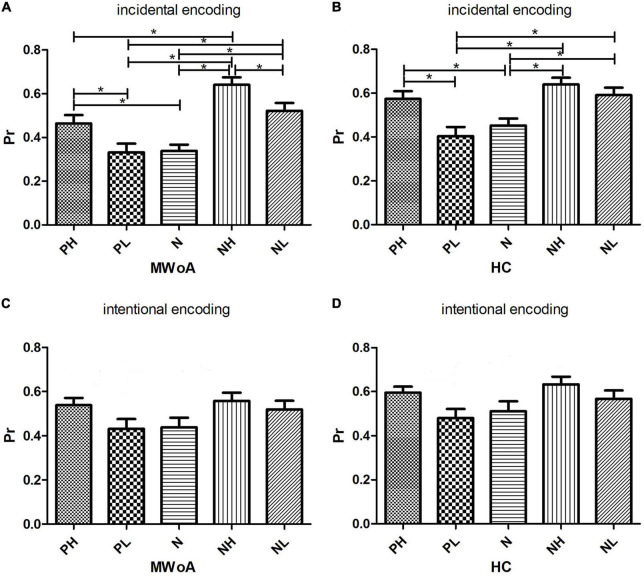
Histograms of simple main effects of emotion on Pr value within the group. **(A,B)** The comparison of Pr between the 5 categories of stimuli under incidental encoding in MWoA patients and HCs, respectively. **(C,D)** The comparison of Pr between the 5 categories of stimuli under intentional encoding in MWoA patients and HCs, respectively. *p < 0.05. Bars represent means ± standard errors.

For incidental encoding, the group x emotion interaction was significant [*F*(4, 188) = 2.449, *p* = 0.048] (Figure 2A). The result suggested a bias in collection performance between the two groups. Then, further analysis showed that the simple main effect of emotion (within-subject factors), but not group (between-subject factors), was significant. The PH, NH, and NL stimuli were better discriminated compared to neutral stimuli in MWoA patients (*p* = 0.01, *p* < 0.001, and *p* = 0.001) and HCs (*p* = 0.023, *p* < 0.001, and *p* = 0.006). Therefore, these results illustrated the EEM effect in MWoA patients and HCs. Moreover, MWoA patients (*p* = 0.009) and HCs (*p* = 0.001) recollected the PH stimuli better than the PL stimuli; however, only the MWoA patients (*p* < 0.001), but not HCs (*p* > 0.05), recollected the NH stimuli better than the NL stimuli. Similarly, the patients (*p* < 0.001) and HCs (*p* < 0.001) recollected the NL stimuli better than the PL stimuli; however, only the patients (*p* < 0.001), but not HCs (*p* > 0.05), recollected the NH stimuli better than PH stimuli. Besides, NH stimuli were better recollected than PL stimuli in both groups (*p* < 0.001) (Figures 3A,B). In brief, the results showed that MWoA patients were more sensitive to emotional stimuli composition (valence and arousal) (Table 2). Besides, no significant main effect of group [*F*(1, 47) = 0.663, *p* = 0.419] or emotion [*F*(4, 188) = 0.933, *p* = 0.446] was observed.

**TABLE 2:** The Pr value of patients with MWoA and healthy controls.

	Incidental encoding		Intentional encoding	
		
	MWoA	HC	MWoA	HC
N	0.338 ± 0.151	0.452 ± 0.161	0.438 ± 0.224	0.511 ± 0.222
PH	0.464 ± 0.200	0.574 ± 0.174	0.538 ± 0.167	0.595 ± 0.137
PL	0.332 ± 0.209	0.403 ± 0.210	0.432 ± 0.224	0.480 ± 0.206
NH	0.641 ± 0.176	0.640 ± 0.150	0.557 ± 0.194	0.633 ± 0.173
NL	0.522 ± 0.187	0.591 ± 0.168	0.519 ± 0.204	0.567 ± 0.191

#### Intentional encoding

For intentional encoding, the main effect of group [*F*(1, 47) = 0.814, *p* = 0.372] or emotion [*F*(4, 188) = 0.144, *p* = 0.965], and the group x emotional interaction [*F*(4, 188) = 0.125, *p* = 0.973] was insignificant (Figures 2B, 3C,D and Table 2).

### Correlation between gray matter volume and recollection performance

**FIGURE 4:**
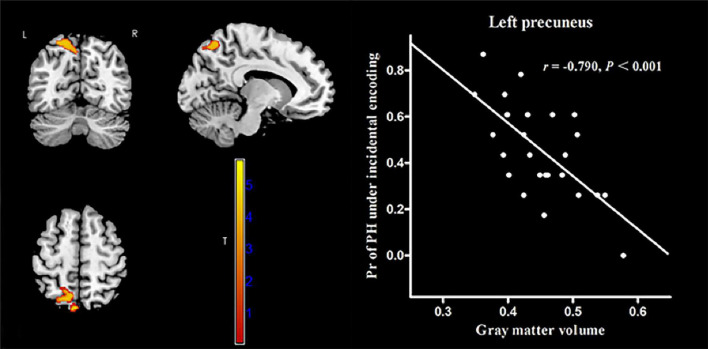
Correlation between voxel-wise gray matter volume in the left precuneus and the Pr-PH (positive stimulus with high arousal) under incidental encoding in MWoA patients. *p* < 0.05, cluster-level FWE corrected. Scatter plot of ROI-based partial correlation analysis between gray matter volume in the left precuneus and the Pr-PH.

**FIGURE 5:**
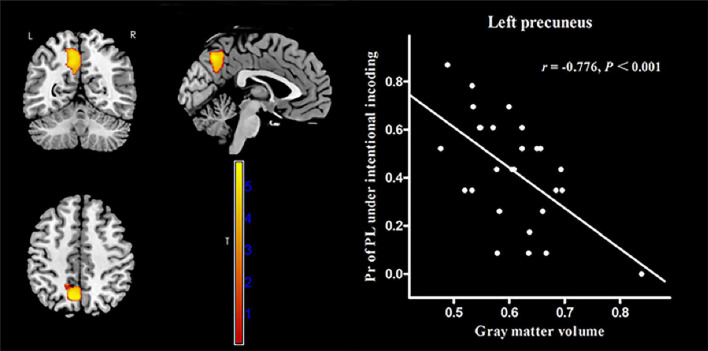
Correlation between voxel-wise gray matter volume in the left precuneus and the Pr-PL (positive stimulus with low arousal) under intentional encoding in MWoA patients. *p* < 0.05, cluster-level FWE corrected. Scatter plot of ROI-based partial correlation analysis between gray matter volume in the left precuneus and the Pr-PL.

In MWoA patients, the GMV of the left precuneus [cluster size = 601 voxels, peak MNI coordinate x/y/z = −6/−78/57, peak *T* = 5.72, and partial correlation coefficient (*r*) = −0.79, *p* < 0.001, Figure 4] was negatively correlated (*p* < 0.05, FWE corrected) with the Pr-PH under incidental encoding. Interestingly, the GMV of another cluster in the left precuneus (cluster size = 1144 voxels, peak MNI coordinate x/y/z = −7.5/−61.5/39, peak *T* = 5.75, and *r* = −0.776, *p* < 0.001, Figure 5) were negatively correlated (*p* < 0.05, FWE corrected) with Pr-PL under intentional encoding. No other significant correlations between the GMV and Pr were observed in MWoA patients. However, no significant correlations between the GMV in the left precuneus and Pr were found for HCs in voxel-wise analysis. Moreover, the GMV of the significant brain region derived using correlation analysis was extracted in MWoA patients, and partial correlation analysis was performed with the Pr in HCs. However, significant results were not identified (*p* > 0.05).

### Correlation between resting state functional connectivity and recollection performance

**FIGURE 6:**
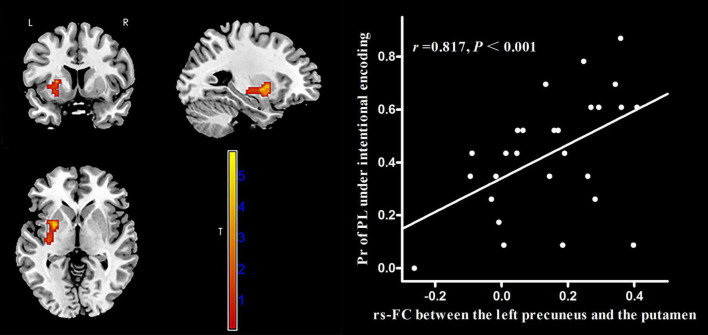
Correlation between voxel-wise rs-FC of the left precuneus and the left putamen and the Pr-PL (positive stimulus with low arousal) under intentional encoding in MWoA patients. *p* < 0.05, cluster-level FWE corrected. Scatter plot of ROI-based partial correlation analysis between rs-FC and the Pr-PL.

The AAL atlas defined the left precuneus as the seed region for rs-FC analysis. In MWoA patients, the rs-FC between the left precuneus and the left putamen (cluster size = 62 voxels, peak MNI coordinate x/y/z = 33/−12/−6, peak *T* = 5.73, and *r* = 0.817, *p* < 0.001, Figure 6) was positively correlated (*p* < 0.05, FWE corrected) with the Pr-PL under intentional encoding. In HCs, the rs-FCs between the left precuneus and several brain regions (the lingual gyrus, calcarine sulcus, superior temporal gyrus, paracentral lobule, and postcentral lobule) were positively correlated (*p* < 0.05, FWE corrected) with the Pr-PH under incidental encoding (Table 3). The rs-FC between the left precuneus and inferior parietal cortex was also positively correlated (*p* < 0.05, FWE corrected) to the Pr-PH under intentional encoding (Table 3).

**TABLE 3:** Correlation between the recollection performance and the rs-FC of the left precuneus and brain regions in healthy controls.

Brian regions	Peak *T*	cluster size	Peak MNI (mm)		
			
			x	y	z
**Incidental encoding pR-PH**					
Lingual gyrus	4.84	115	−15	−87	−18
Calcarine	5.17	116	18	−96	9
Superior temporal	6.03	93	−51	−21	6
Paracentral lobule	5.57	196	−6	−42	53
Postcentral	5.42	61	24	−30	57
**Intentional encoding pR-PH**					
Inferior parietal	5.93	93	−42	−60	51

*p* < 0.05, cluster level FWE corrected. Coordinates of peak voxels (x, y, z) are given in MNI space.

## Discussion

Recollection and familiarity are two separate processes underlying emotional memory. Based on the dual-process approach, familiarity reflects a classical signal-detection process, and recollection reflects a threshold process (Yonelinas, 2001). The recollection, but not familiarity, decreases over time, particularly over short intervals, and the decline of the recollection component causes memory loss (Kishiyama et al., 2005; Yang et al., 2016). Therefore, the analysis focused on the recollection component of emotional memory. In this study, the MWoA patients and HCs exhibited an EEM effect under incidental encoding. Recollection benefited from extreme valence and arousal and increased the distinction during the encoding step (Alonso et al., 2015). Kensinger et al. (2007) demonstrated a similar enhancement for negative and high arousal stimuli compared to neutral ones. Under intentional encoding, both groups’ EEM effect was missing, consistent with previous reports in young adults (Kensinger et al., 2005, 2007). For intentional encoding, participants attempted to focus their cognitive resources on all memorization items, including neutral stimulus, by prompting subsequent retrieval tasks. For incidental encoding, participants were uninformed of subsequent retrieval tasks; therefore, they could not allocate similar resources, resulting in a subjective connection toward different stimuli/items. MWoA patients might be benefiting from the intentional encoding instructions and elaborate encoding strategies, similarly to the HCs (Kensinger et al., 2005). Understanding the enhancement of emotional characteristics on memory in MWoA patients could help develop interventions to prevent dementia and promote healthy aging (Alonso et al., 2015). Notably, MWoA patients exhibited better recollection performance for PH than NH and NH than NL under incidental encoding. These differences were not observed in HCs, indicating that patients with MWoA were more susceptible to valence and arousal of emotional stimuli. However, we did not find a deterioration in recollection performance or EEM effect. The reasons for this are likely twofold. On the one hand, the limbic circuitry, comprised of the amygdala and hippocampus, is not only responsible for emotions, learning, and memory but is also implicated in experiential aspects of pain. It has been demonstrated that the chronification of pain was activity-induced plasticity of limbic cortical circuits leading to neocortex reorganization, during which the representation of pain gradually shifts from sensory to the memory of pain and/or the inability to extinguish painful memories (McCarberg and Peppin, 2019). One the other hand, previous studies reported that MWoA patients showed enhanced brain activation toward emotional stimulation (Wilcox et al., 2016) and were more sensitive to negative stimuli (Wang et al., 2017). In particular, the unpleasant category of IAPS images included highly arousing mutilation and attack images (Lang et al., 2010). Although poor global cognitive functions were observed in migraine patients, they may not capture changes in general intellectual functions or might be sensitive to changes in specific cognitive domains (Gates et al., 2019). Thus, a tendency for influence of MWoA on emotional memory was considered.

In MWoA patients, we observed correlations between the GMV of the left precuneus and recollection performance. However, the precuneus is a core region of the default mode network (DMN) involved in episodic memory (Buckner et al., 2008). The abnormalities in the precuneus might affect information transfer, multimodal integration, and pain sensitivity and processing in MWoA patients (Zhang et al., 2016). The left precuneus might be damaged, remodeled, and involved in a compensation mechanism during pain management, which might explain the negative correlation between the GMV of the left precuneus and recollection performance. Moreover, the rs-FC between the left precuneus and the left putamen positively correlated with the recollection performance. The putamen is involved in cognitive, emotional, and reward processing (Haber and Knutson, 2010; Ghandili and Munakomi, 2022) and connects to the components of the DMN (Byrne et al., 2019). In this study, the FC of this intra-reward system was correlated with the recollection performance in MWoA patients.

GMV in the left precuneus and the rs-FC between the left precuneus and the left putamen were not correlated to the recollection performance in HCs. The precuneus, putamen, and reward systems participate in pain processing during migraine (Tanasescu et al., 2016; Zhang et al., 2016; Porreca and Navratilova, 2017). Therefore, we believed these associations might result from the pathological mechanism of the disease itself. In HCs, the rs-FCs between the left precuneus and several brain regions (the lingual gyrus, calcarine sulcus, superior temporal gyrus, paracentral lobule, and postcentral lobule) were positively correlated with recollection performance. The lingual gyrus and calcarine sulcus belong to the visual cortex, (Huff et al., 2022). Gao et al. (2019) revealing that the superior temporal gyrus was a brain region responsible for audiovisual affective and emotional processing. Moreover, the inferior parietal lobule is a component of the ventral attention network (Corbetta et al., 2008). It indicated that information from various brain areas/networks was integrated to successfully perform the task in HCs. When patients are exposed to a migraine attack, changes in neurobiological progression might lead to changes in mood, vision, attention, cognition, and behavioral regulation. The integration of the necessary information for behavior might be disturbed. However, neural reserve and compensation support the cognitive reserve (Anthony and Lin, 2018). In MWoA patients, the performance of emotional memory might be maintained because of compensatory contribution from the left precuneus.

This study had several limitations. First, it was a cross-sectional study with a sex-skewed sample and a widely spread age group. The included migraine patients were only without aura, and their education level was relatively high. Longitudinal studies with expanding sample diversity, such as migraine subtypes and left-hand participants, are needed in the future to observe the applicability of the current results. Second, although we controlled for several potential confounders, residual confounding factors could exist. Third, the behavioral test for emotional memory was within a laboratory setting, and tests are required in real-life environments in the future. Moreover, the recollection performance may differ when using different encoding-retrieval interval times (Yang et al., 2016). Finally, for the rs-FC analyses, the left precuneus was focused on and set as the seed region. Other brain regions should also be incorporated in future studies.

## Conclusion

In conclusion, our results revealed the tendency for the influence of migraine on emotional memory. Although these impacts were insufficient to indicate deterioration in recollection performance and EEM effect, the patients with MWoA were more sensitive to valence and arousal of emotional stimuli under incidental encoding. Moreover, a difference was found in the structural and functional contributions of the left precuneus in recollection performance between MWoA patients and HCs, indicating its crucial role in emotional memory. These findings help recognize the association between migraine and emotional memory and its neural correlations. It might provide novel insights into early interventions in preventing cognitive decline because of migraine.

## Data availability statement

The raw data supporting the conclusions of this article will be made available by the authors, without undue reservation.

## Ethics statement

The studies involving human participants were reviewed and approved by the Institutional Review Board and Ethics Committee of the First Affiliated Hospital of Anhui Medical University. The patients/participants provided their written informed consent to participate in this study.

## Author contributions

YY conceived and designed the study. ML and XL drafted and revised the manuscript. WZ and ZG participated in data collection, cognitive function assessment, and visualization. JZ and HW helped with data analysis. XW, SZ, and KW participated in case collection and scale assessment. All authors contributed to the article and approved the submitted version.

## Footnotes

1
https://neuro-jena.github.io/software.html#vbm

2
http://www.fil.ion.ucl.ac.uk/spm/

3
https://rfmri.org/dpabi

4
http://www.alivelearn.net/xjview

## Abbreviations

MWoA: migraine without aura
EEM: emotional enhancement of memory
NH: negative pictures with high arousal
PH: positive pictures with high arousal
NL: negative pictures with low arousal
PL: positive pictures with low arousal
N: neutral pictures
Pr: recollection performance
GMV: gray matter volume
rs-FC: resting-state functional connectivity
MoCA: Montreal Cognitive Assessment
HAMA: Hamilton Anxiety Scale
HAMD: Hamilton Depression Scale.
